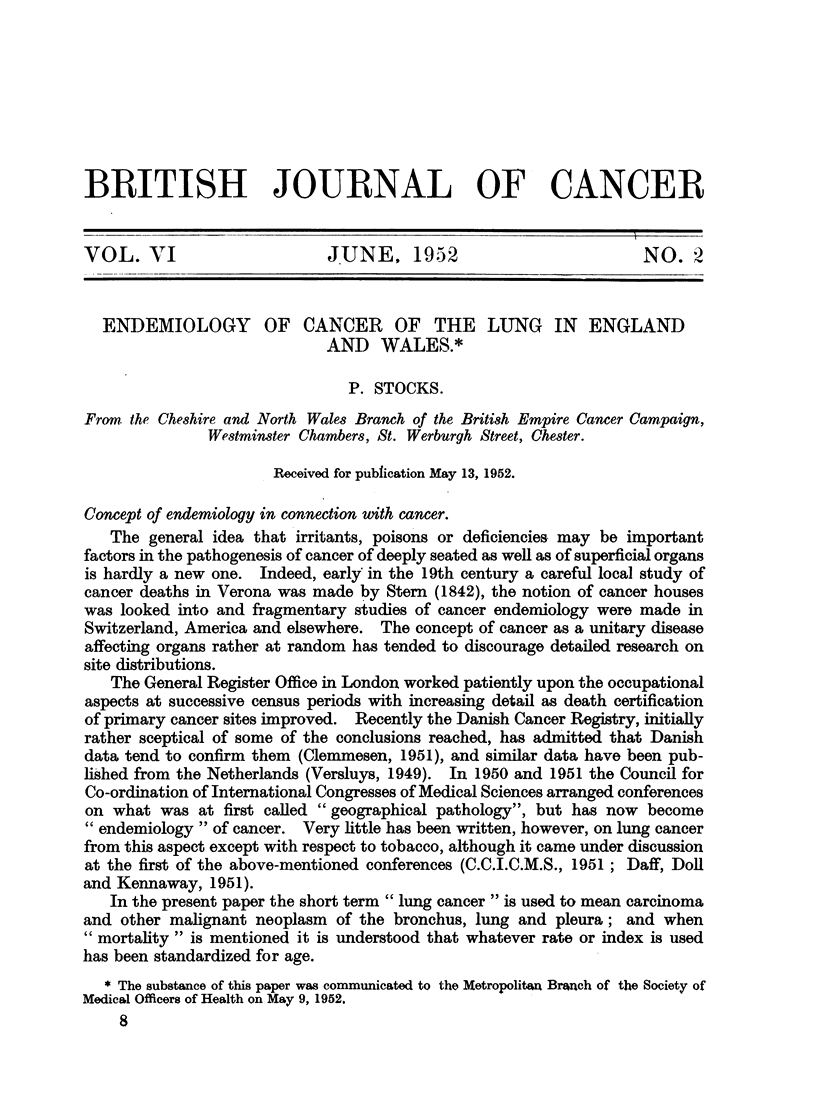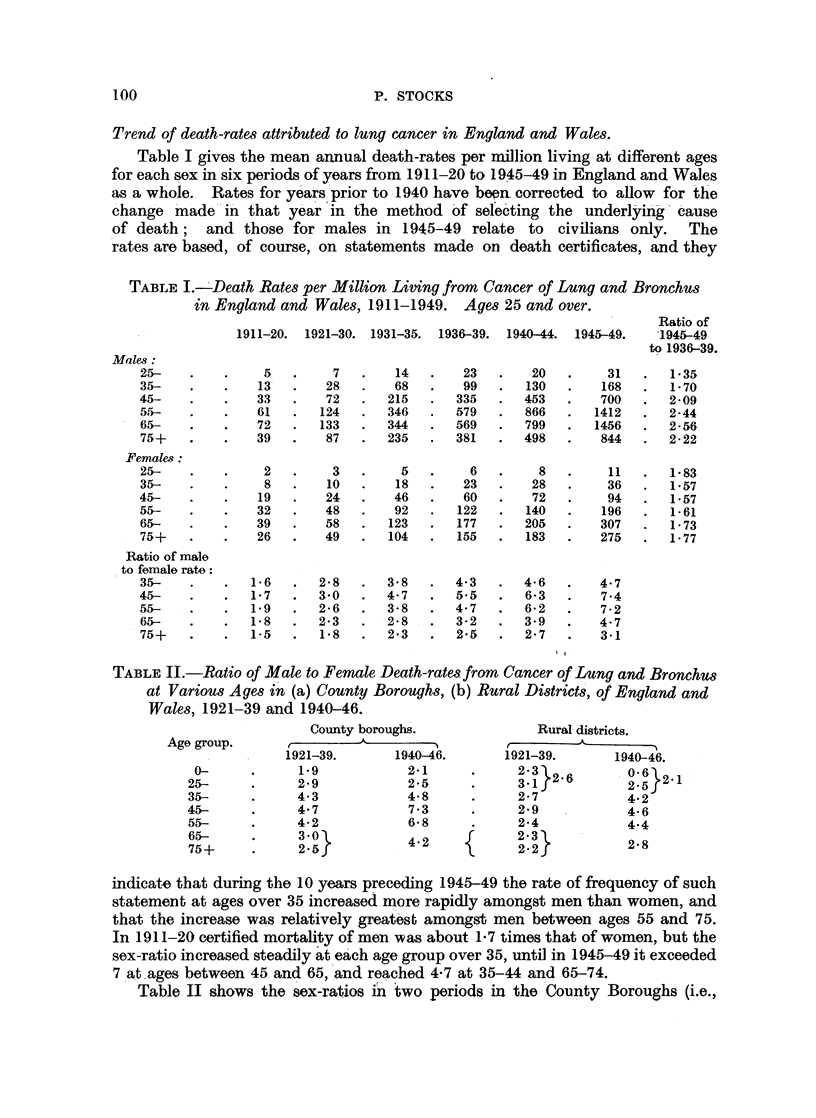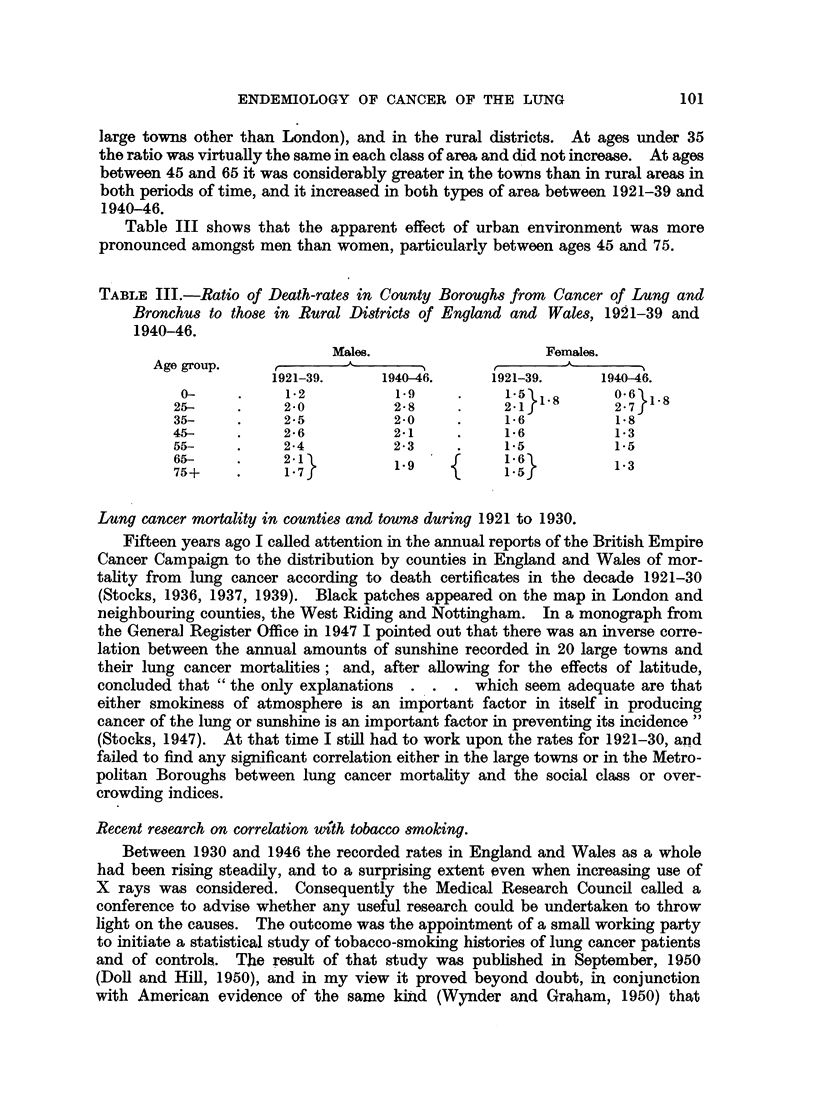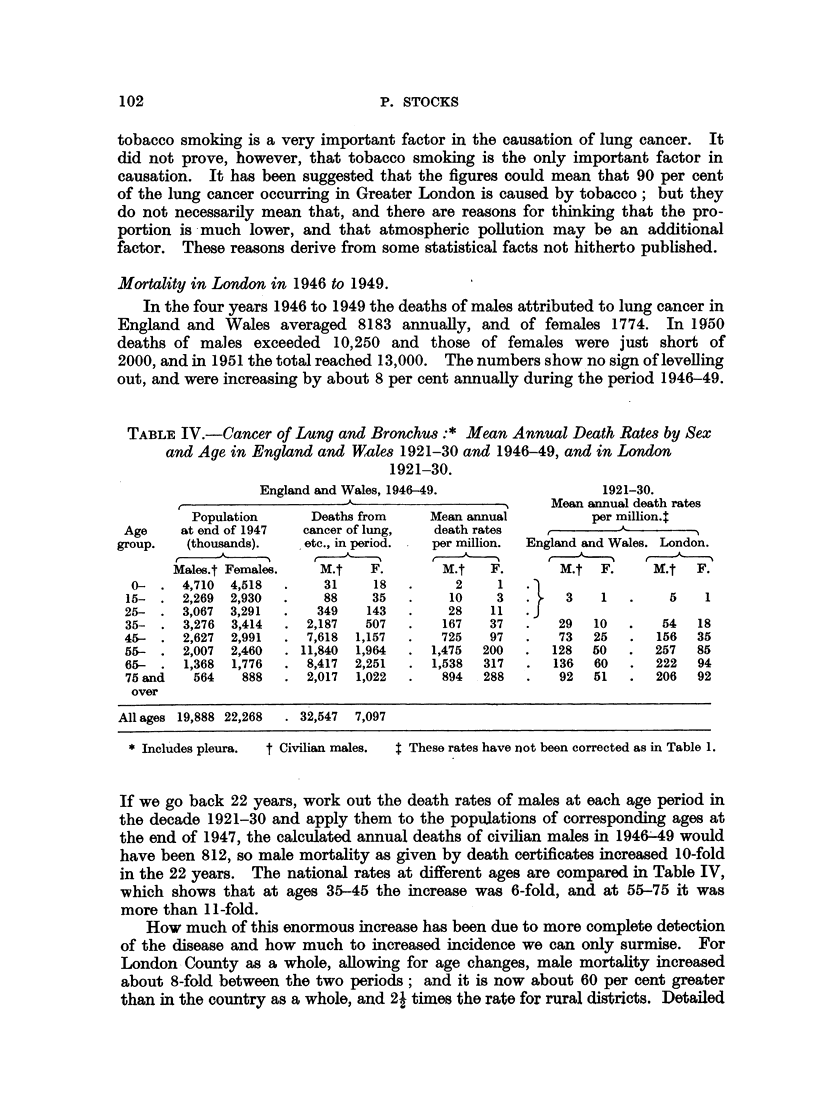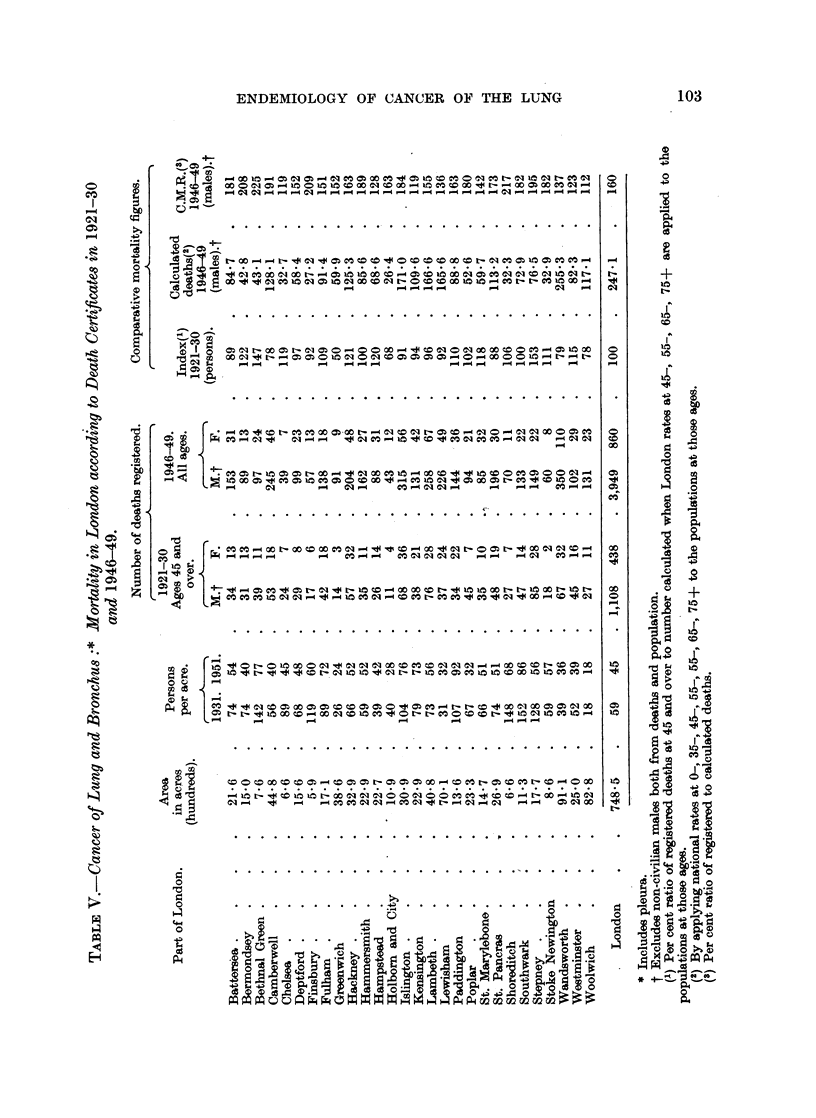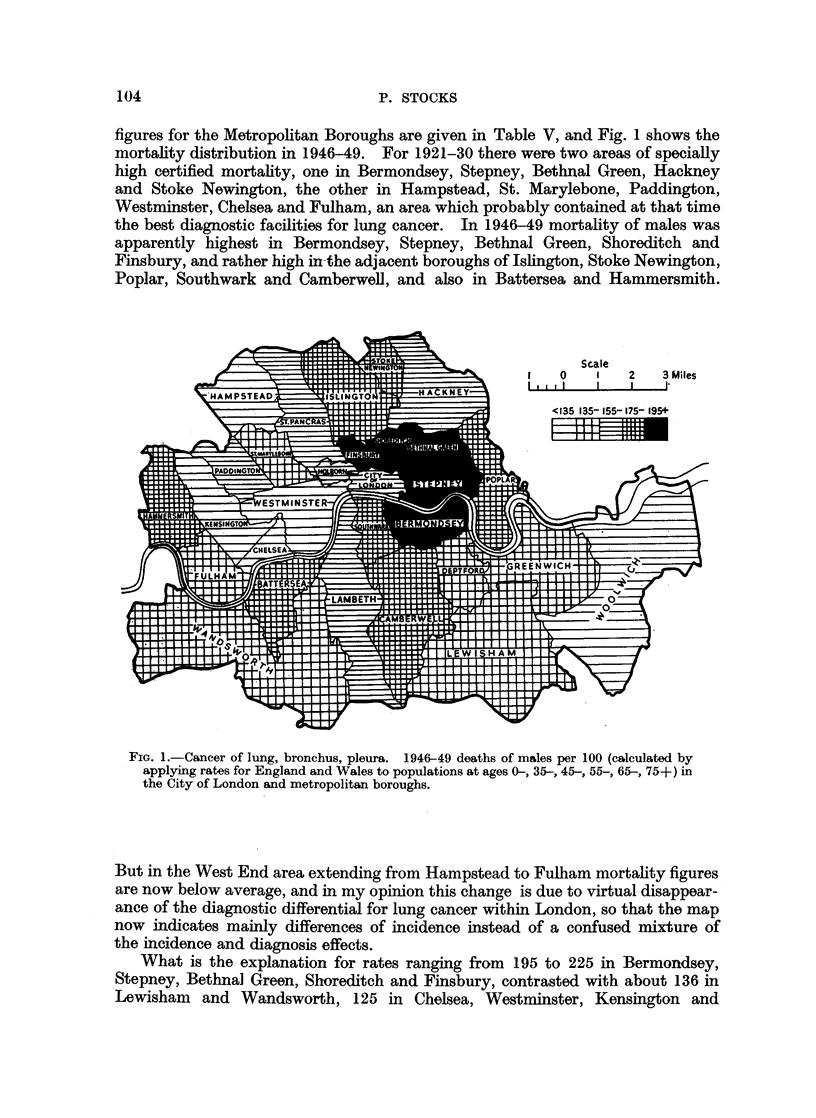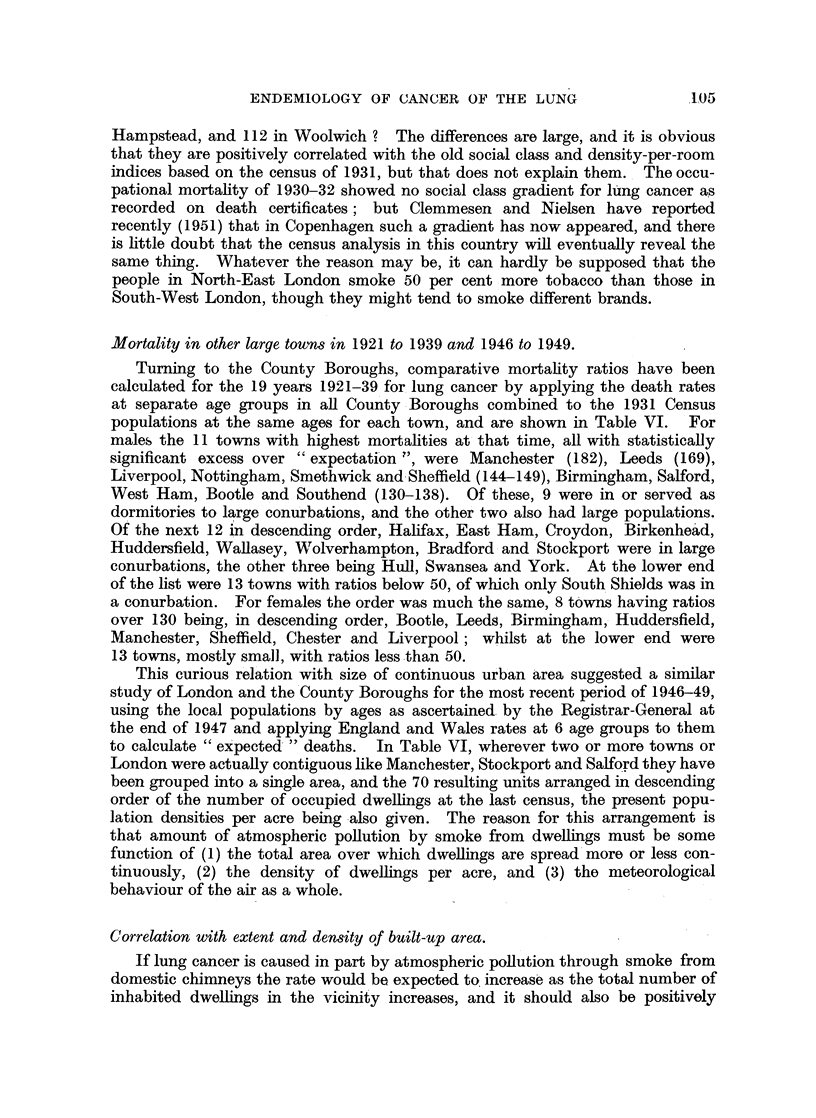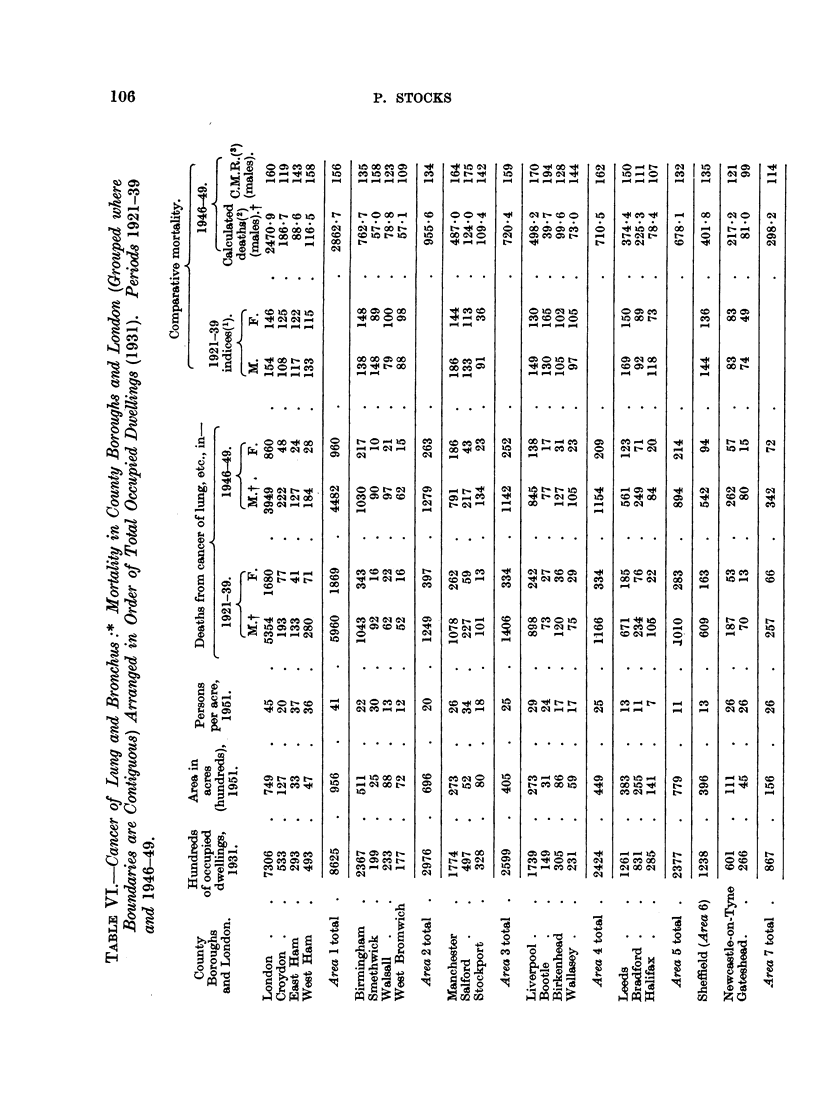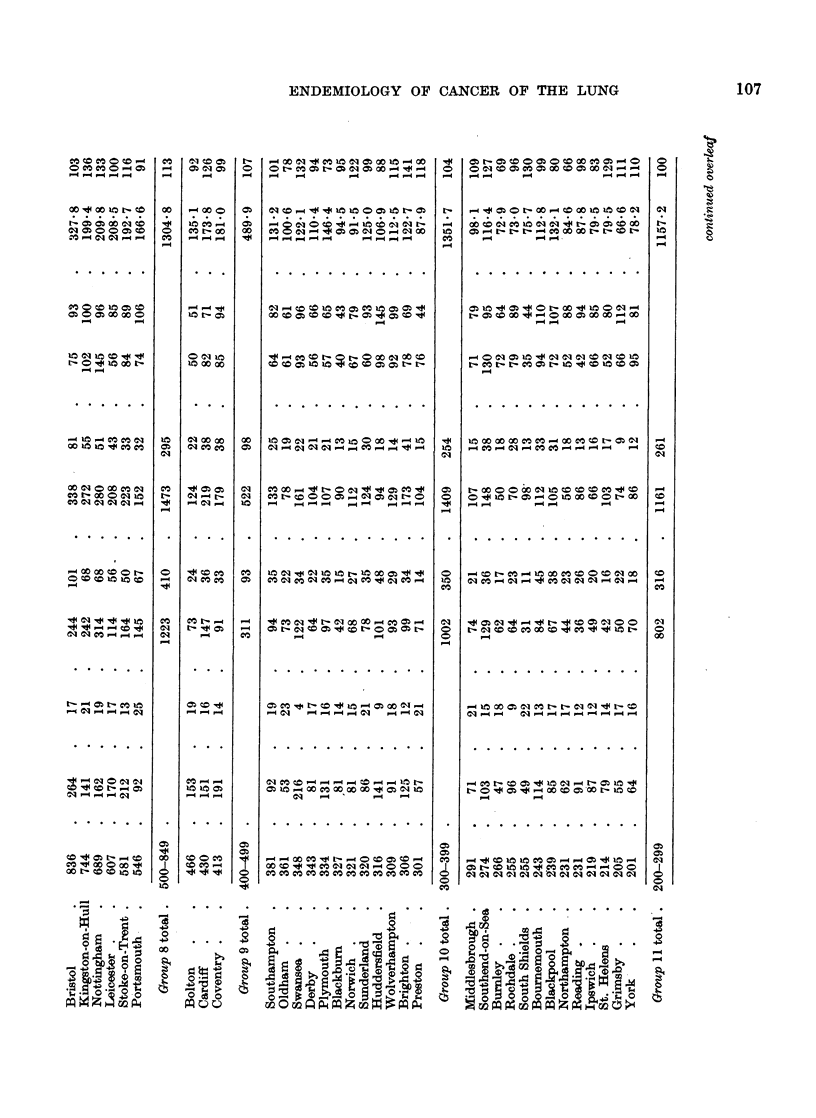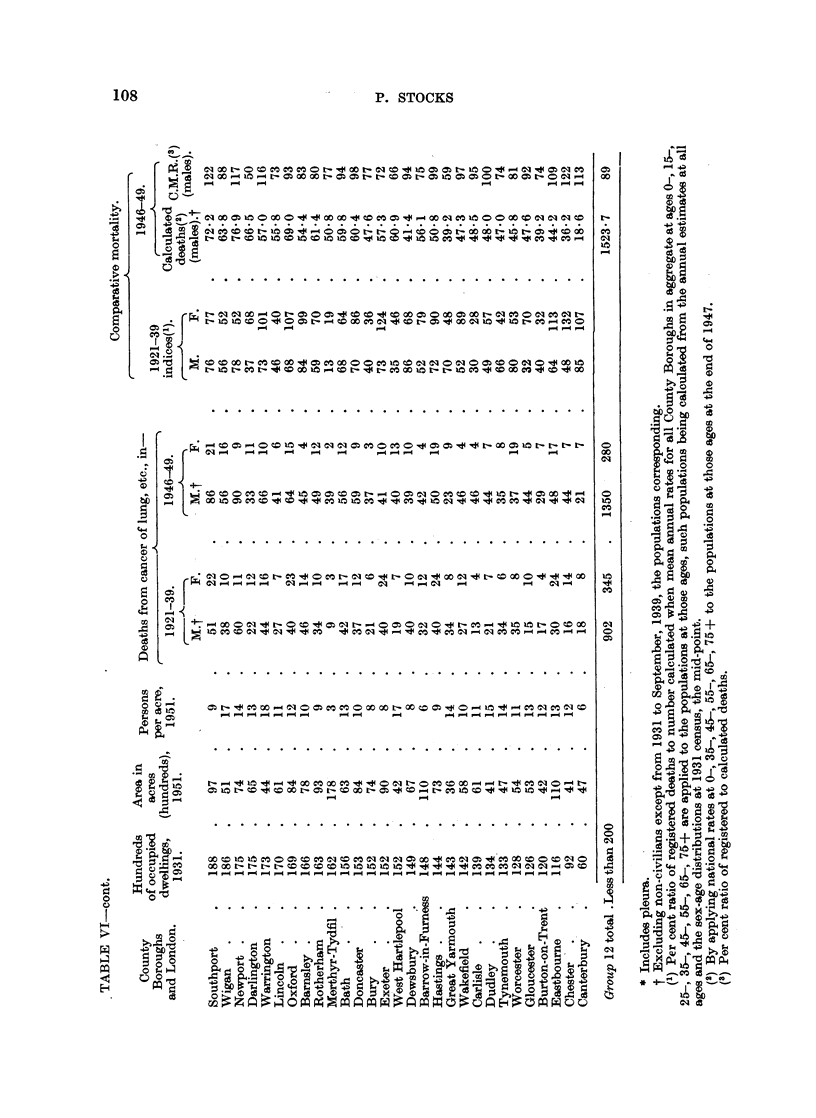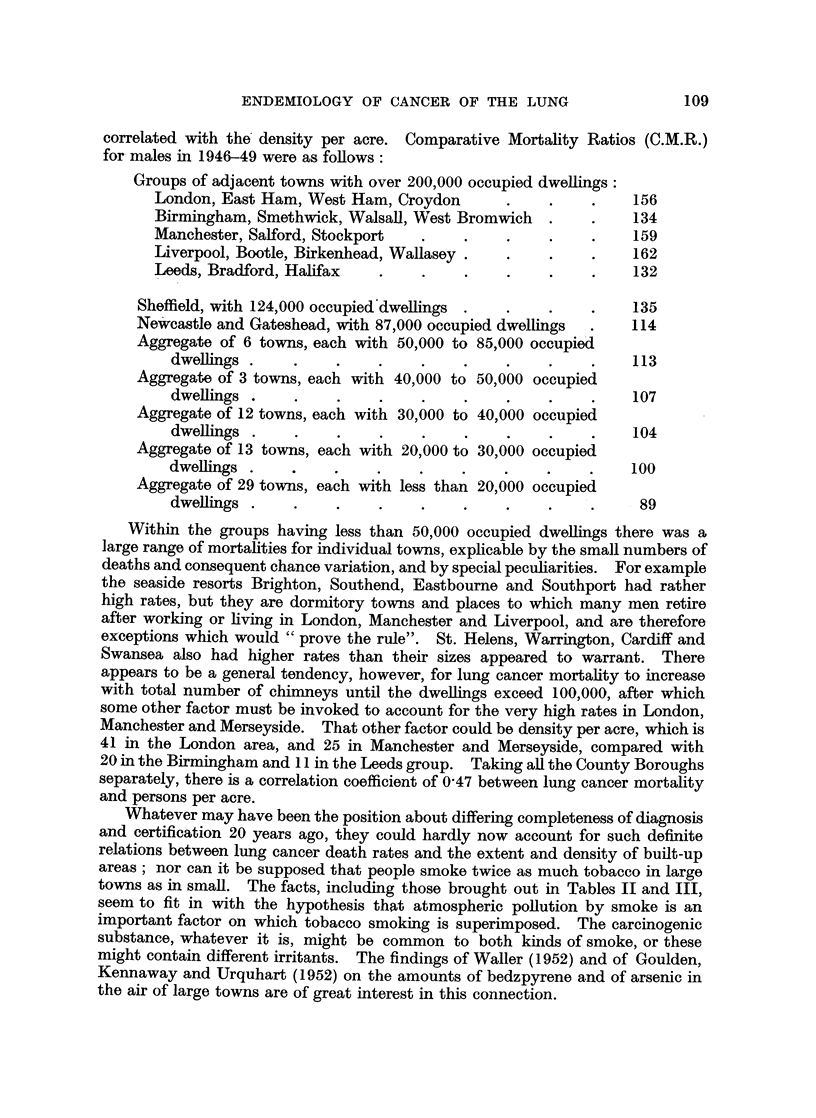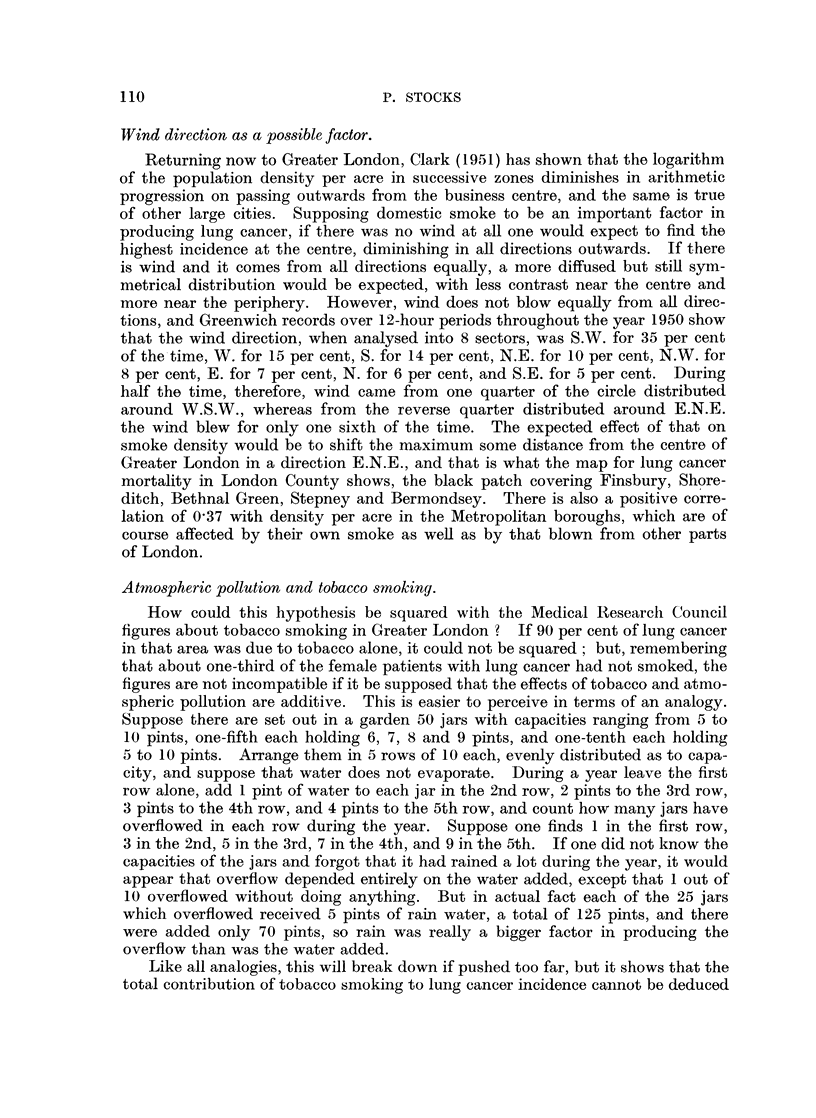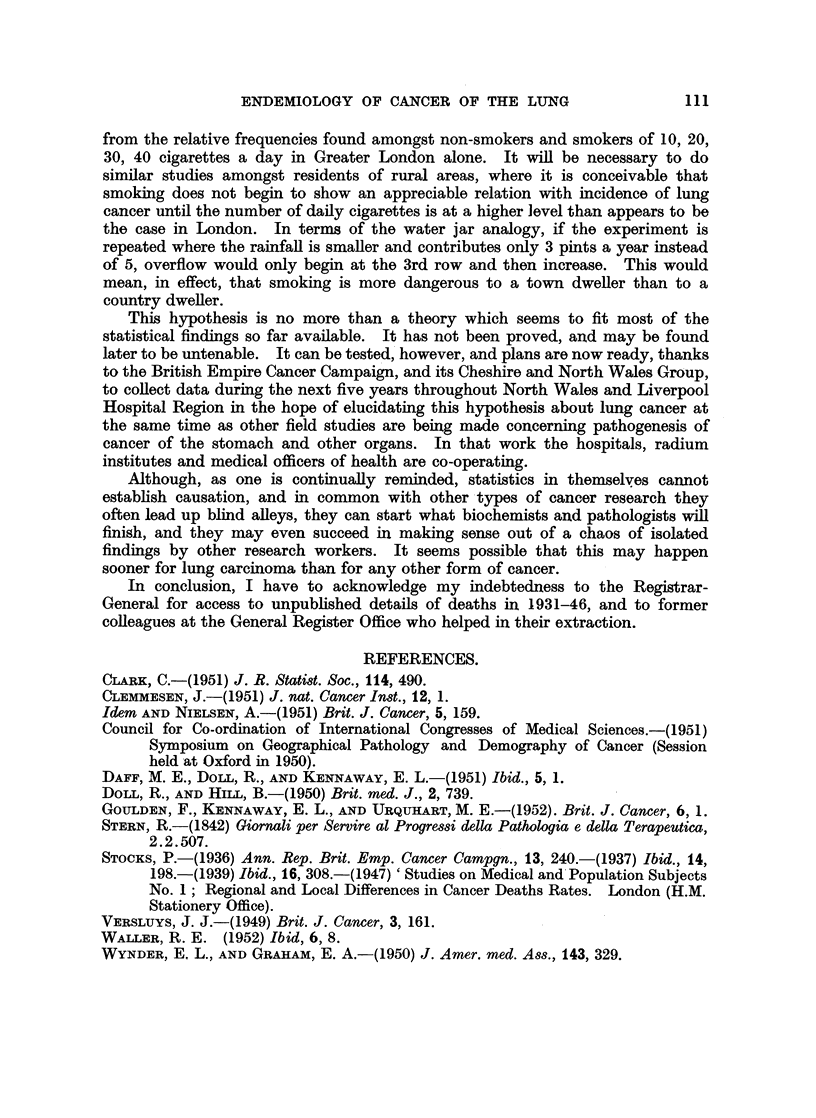# Endemiology of Cancer of the Lung in England and Wales*

**DOI:** 10.1038/bjc.1952.11

**Published:** 1952-06

**Authors:** P. Stocks


BRITISH JOURNAL OF CANCER

VOL. VI JUNE, 1952 NO. 2

### ENDEMIOLOGY OF CANCER OF THE LUNG IN ENGLAND AND WALES.*

P. STOCKS.

*From the Cheshire and North Wales Branch of the British Empire Cancer Campaign, Westminster Chambers, St. Werburgh Street, Chester.*

Received for publication May 13, 1952.

*Concept of endemiology in connection with cancer.*

The general idea that irritants, poisons or deficiencies may be important factors in the pathogenesis of cancer of deeply seated as well as of superficial organs is hardly a new one. Indeed, early in the 19th century a careful local study of cancer deaths in Verona was made by Stern (1842), the notion of cancer houses was looked into and fragmentary studies of cancer endemiology were made in Switzerland, America and elsewhere. The concept of cancer as a unitary disease affecting organs rather at random has tended to discourage detailed research on site distributions.

The General Register Office in London worked patiently upon the occupational aspects at successive census periods with increasing detail as death certification of primary cancer sites improved. Recently the Danish Cancer Registry, initially rather sceptical of some of the conclusions reached, has admitted that Danish data tend to confirm them (Clemmesen, 1951), and similar data have been published from the Netherlands (Versluys, 1949). In 1950 and 1951 the Council for Co-ordination of International Congresses of Medical Sciences arranged conferences on what was at first called "geographical pathology", but has now become "endemiology" of cancer. Very little has been written, however, on lung cancer from this aspect except with respect to tobacco, although it came under discussion at the first of the above-mentioned conferences (C.C.I.C.M.S., 1951 ; Daff, Doll and Kennaway, 1951).

In the present paper the short term "lung cancer" is used to mean carcinoma and other malignant neoplasm of the bronchus, lung and pleura ; and when "mortality" is mentioned it is understood that whatever rate or index is used has been standardized for age.

* The substance of this paper was communicated to the Metropolitan Branch of the Society of Medical Officers of Health on May 9, 1952.

*Trend of death-rates attributed to lung cancer in England and Wales.*

Table I gives the mean annual death-rates per million living at different ages for each sex in six periods of years from 1911–20 to 1945–49 in England and Wales as a whole. Rates for years prior to 1940 have been corrected to allow for the change made in that year in the method of selecting the underlying cause of death; and those for males in 1945–49 relate to civilians only. The rates are based, of course, on statements made on death certificates, and they indicate that during the 10 years preceding 1945–49 the rate of frequency of such statement at ages over 35 increased more rapidly amongst men than women, and that the increase was relatively greatest amongst men between ages 55 and 75. In 1911–20 certified mortality of men was about 1·7 times that of women, but the sex-ratio increased steadily at each age group over 35, until in 1945–49 it exceeded 7 at ages between 45 and 65, and reached 4·7 at 35–44 and 65–74.

TABLE I.—*Death Rates per Million Living from Cancer of Lung and Bronchus in England and Wales,* 1911–1949. *Ages* 25 *and over.*

| | 1911–20. | 1921–30. | 1931–35. | 1936–39. | 1940–44. | 1945–49. | Ratio of 1945–49 to 1936–39. |
|---|---|---|---|---|---|---|---|
| *Males :* | | | | | | | |
| 25– | 5 | 7 | 14 | 23 | 20 | 31 | 1·35 |
| 35– | 13 | 28 | 68 | 99 | 130 | 168 | 1·70 |
| 45– | 33 | 72 | 215 | 335 | 453 | 700 | 2·09 |
| 55– | 61 | 124 | 346 | 579 | 866 | 1412 | 2·44 |
| 65– | 72 | 133 | 344 | 569 | 799 | 1456 | 2·56 |
| 75+ | 39 | 87 | 235 | 381 | 498 | 844 | 2·22 |
| *Females :* | | | | | | | |
| 25– | 2 | 3 | 5 | 6 | 8 | 11 | 1·83 |
| 35– | 8 | 10 | 18 | 23 | 28 | 36 | 1·57 |
| 45– | 19 | 24 | 46 | 60 | 72 | 94 | 1·57 |
| 55– | 32 | 48 | 92 | 122 | 140 | 196 | 1·61 |
| 65– | 39 | 58 | 123 | 177 | 205 | 307 | 1·73 |
| 75+ | 26 | 49 | 104 | 155 | 183 | 275 | 1·77 |
| Ratio of male to female rate : | | | | | | | |
| 35– | 1·6 | 2·8 | 3·8 | 4·3 | 4·6 | 4·7 | |
| 45– | 1·7 | 3·0 | 4·7 | 5·5 | 6·3 | 7·4 | |
| 55– | 1·9 | 2·6 | 3·8 | 4·7 | 6·2 | 7·2 | |
| 65– | 1·8 | 2·3 | 2·8 | 3·2 | 3·9 | 4·7 | |
| 75+ | 1·5 | 1·8 | 2·3 | 2·5 | 2·7 | 3·1 | |

TABLE II.—*Ratio of Male to Female Death-rates from Cancer of Lung and Bronchus at Various Ages in* (a) *County Boroughs,* (b) *Rural Districts, of England and Wales,* 1921–39 and 1940–46.

| Age group. | County boroughs. | | Rural districts. | |
|---|---|---|---|---|
| | 1921–39. | 1940–46. | 1921–39. | 1940–46. |
| 0– | 1·9 | 2·1 | 2·3 } 2·6 | 0·6 } 2·1 |
| 25– | 2·9 | 2·5 | 3·1 } | 2·5 } |
| 35– | 4·3 | 4·8 | 2·7 | 4·2 |
| 45– | 4·7 | 7·3 | 2·9 | 4·6 |
| 55– | 4·2 | 6·8 | 2·4 | 4·4 |
| 65– | 3·0 } | 4·2 | 2·3 } | 2·8 |
| 75+ | 2·5 } | | 2·2 } | |

Table II shows the sex-ratios in two periods in the County Boroughs (i.e., large towns other than London), and in the rural districts. At ages under 35 the ratio was virtually the same in each class of area and did not increase. At ages between 45 and 65 it was considerably greater in the towns than in rural areas in both periods of time, and it increased in both types of area between 1921–39 and 1940–46.

Table III shows that the apparent effect of urban environment was more pronounced amongst men than women, particularly between ages 45 and 75.

TABLE III.—*Ratio of Death-rates in County Boroughs from Cancer of Lung and Bronchus to those in Rural Districts of England and Wales,* 1921–39 and 1940–46.

| Age group. | Males. 1921–39. | Males. 1940–46. | Females. 1921–39. | Females. 1940–46. |
|---|---|---|---|---|
| 0– | 1·2 | 1·9 | 1·5 } 1·8 | 0·6 } 1·8 |
| 25– | 2·0 | 2·8 | 2·1 | 2·7 |
| 35– | 2·5 | 2·0 | 1·6 | 1·8 |
| 45– | 2·6 | 2·1 | 1·6 | 1·3 |
| 55– | 2·4 | 2·3 | 1·5 | 1·5 |
| 65– | 2·1 } | 1·9 | 1·6 } | 1·3 |
| 75+ | 1·7 } | | 1·5 } | |

*Lung cancer mortality in counties and towns during* 1921 to 1930.

Fifteen years ago I called attention in the annual reports of the British Empire Cancer Campaign to the distribution by counties in England and Wales of mortality from lung cancer according to death certificates in the decade 1921–30 (Stocks, 1936, 1937, 1939). Black patches appeared on the map in London and neighbouring counties, the West Riding and Nottingham. In a monograph from the General Register Office in 1947 I pointed out that there was an inverse correlation between the annual amounts of sunshine recorded in 20 large towns and their lung cancer mortalities ; and, after allowing for the effects of latitude, concluded that " the only explanations . . . which seem adequate are that either smokiness of atmosphere is an important factor in itself in producing cancer of the lung or sunshine is an important factor in preventing its incidence " (Stocks, 1947). At that time I still had to work upon the rates for 1921–30, and failed to find any significant correlation either in the large towns or in the Metropolitan Boroughs between lung cancer mortality and the social class or overcrowding indices.

*Recent research on correlation with tobacco smoking.*

Between 1930 and 1946 the recorded rates in England and Wales as a whole had been rising steadily, and to a surprising extent even when increasing use of X rays was considered. Consequently the Medical Research Council called a conference to advise whether any useful research could be undertaken to throw light on the causes. The outcome was the appointment of a small working party to initiate a statistical study of tobacco-smoking histories of lung cancer patients and of controls. The result of that study was published in September, 1950 (Doll and Hill, 1950), and in my view it proved beyond doubt, in conjunction with American evidence of the same kind (Wynder and Graham, 1950) that tobacco smoking is a very important factor in the causation of lung cancer. It did not prove, however, that tobacco smoking is the only important factor in causation. It has been suggested that the figures could mean that 90 per cent of the lung cancer occurring in Greater London is caused by tobacco; but they do not necessarily mean that, and there are reasons for thinking that the proportion is much lower, and that atmospheric pollution may be an additional factor. These reasons derive from some statistical facts not hitherto published.

*Mortality in London in* 1946 *to* 1949.

In the four years 1946 to 1949 the deaths of males attributed to lung cancer in England and Wales averaged 8183 annually, and of females 1774. In 1950 deaths of males exceeded 10,250 and those of females were just short of 2000, and in 1951 the total reached 13,000. The numbers show no sign of levelling out, and were increasing by about 8 per cent annually during the period 1946–49.

TABLE IV.—*Cancer of Lung and Bronchus:* Mean Annual Death Rates by Sex and Age in England and Wales* 1921–30 *and* 1946–49, *and in London* 1921–30.

| Age group. | England and Wales, 1946–49. Population at end of 1947 (thousands). Males.† | Females. | Deaths from cancer of lung, etc., in period. M.† | F. | Mean annual death rates per million. M.† | F. | 1921–30. Mean annual death rates per million.‡ England and Wales. M.† | F. | London. M.† | F. |
|---|---|---|---|---|---|---|---|---|---|---|
| 0– | 4,710 | 4,518 | 31 | 18 | 2 | 1 | | | | |
| 15– | 2,269 | 2,930 | 88 | 35 | 10 | 3 | 3 | 1 | 5 | 1 |
| 25– | 3,067 | 3,291 | 349 | 143 | 28 | 11 | | | | |
| 35– | 3,276 | 3,414 | 2,187 | 507 | 167 | 37 | 29 | 10 | 54 | 18 |
| 45– | 2,627 | 2,991 | 7,618 | 1,157 | 725 | 97 | 73 | 25 | 156 | 35 |
| 55– | 2,007 | 2,460 | 11,840 | 1,964 | 1,475 | 200 | 128 | 50 | 257 | 85 |
| 65– | 1,368 | 1,776 | 8,417 | 2,251 | 1,538 | 317 | 136 | 60 | 222 | 94 |
| 75 and over | 564 | 888 | 2,017 | 1,022 | 894 | 288 | 92 | 51 | 206 | 92 |
| All ages | 19,888 | 22,268 | 32,547 | 7,097 | | | | | | |

* Includes pleura. † Civilian males. ‡ These rates have not been corrected as in Table 1.

If we go back 22 years, work out the death rates of males at each age period in the decade 1921–30 and apply them to the populations of corresponding ages at the end of 1947, the calculated annual deaths of civilian males in 1946–49 would have been 812, so male mortality as given by death certificates increased 10-fold in the 22 years. The national rates at different ages are compared in Table IV, which shows that at ages 35–45 the increase was 6-fold, and at 55–75 it was more than 11-fold.

How much of this enormous increase has been due to more complete detection of the disease and how much to increased incidence we can only surmise. For London County as a whole, allowing for age changes, male mortality increased about 8-fold between the two periods; and it is now about 60 per cent greater than in the country as a whole, and $2\frac{1}{2}$ times the rate for rural districts. Detailed figures for the Metropolitan Boroughs are given in Table V, and Fig. 1 shows the mortality distribution in 1946–49. For 1921–30 there were two areas of specially high certified mortality, one in Bermondsey, Stepney, Bethnal Green, Hackney and Stoke Newington, the other in Hampstead, St. Marylebone, Paddington, Westminster, Chelsea and Fulham, an area which probably contained at that time the best diagnostic facilities for lung cancer. In 1946–49 mortality of males was apparently highest in Bermondsey, Stepney, Bethnal Green, Shoreditch and Finsbury, and rather high in the adjacent boroughs of Islington, Stoke Newington, Poplar, Southwark and Camberwell, and also in Battersea and Hammersmith.

FIG. 1.—Cancer of lung, bronchus, pleura. 1946–49 deaths of males per 100 (calculated by applying rates for England and Wales to populations at ages 0–, 35–, 45–, 55–, 65–, 75+) in the City of London and metropolitan boroughs.

But in the West End area extending from Hampstead to Fulham mortality figures are now below average, and in my opinion this change is due to virtual disappearance of the diagnostic differential for lung cancer within London, so that the map now indicates mainly differences of incidence instead of a confused mixture of the incidence and diagnosis effects.

What is the explanation for rates ranging from 195 to 225 in Bermondsey, Stepney, Bethnal Green, Shoreditch and Finsbury, contrasted with about 136 in Lewisham and Wandsworth, 125 in Chelsea, Westminster, Kensington and

Hampstead, and 112 in Woolwich ? The differences are large, and it is obvious that they are positively correlated with the old social class and density-per-room indices based on the census of 1931, but that does not explain them. The occupational mortality of 1930–32 showed no social class gradient for lung cancer as recorded on death certificates ; but Clemmesen and Nielsen have reported recently (1951) that in Copenhagen such a gradient has now appeared, and there is little doubt that the census analysis in this country will eventually reveal the same thing. Whatever the reason may be, it can hardly be supposed that the people in North-East London smoke 50 per cent more tobacco than those in South-West London, though they might tend to smoke different brands.

*Mortality in other large towns in* 1921 *to* 1939 *and* 1946 *to* 1949.

Turning to the County Boroughs, comparative mortality ratios have been calculated for the 19 years 1921–39 for lung cancer by applying the death rates at separate age groups in all County Boroughs combined to the 1931 Census populations at the same ages for each town, and are shown in Table VI. For males the 11 towns with highest mortalities at that time, all with statistically significant excess over " expectation ", were Manchester (182), Leeds (169), Liverpool, Nottingham, Smethwick and Sheffield (144–149), Birmingham, Salford, West Ham, Bootle and Southend (130–138). Of these, 9 were in or served as dormitories to large conurbations, and the other two also had large populations. Of the next 12 in descending order, Halifax, East Ham, Croydon, Birkenhead, Huddersfield, Wallasey, Wolverhampton, Bradford and Stockport were in large conurbations, the other three being Hull, Swansea and York. At the lower end of the list were 13 towns with ratios below 50, of which only South Shields was in a conurbation. For females the order was much the same, 8 towns having ratios over 130 being, in descending order, Bootle, Leeds, Birmingham, Huddersfield, Manchester, Sheffield, Chester and Liverpool ; whilst at the lower end were 13 towns, mostly small, with ratios less than 50.

This curious relation with size of continuous urban area suggested a similar study of London and the County Boroughs for the most recent period of 1946–49, using the local populations by ages as ascertained by the Registrar-General at the end of 1947 and applying England and Wales rates at 6 age groups to them to calculate " expected " deaths. In Table VI, wherever two or more towns or London were actually contiguous like Manchester, Stockport and Salford they have been grouped into a single area, and the 70 resulting units arranged in descending order of the number of occupied dwellings at the last census, the present population densities per acre being also given. The reason for this arrangement is that amount of atmospheric pollution by smoke from dwellings must be some function of (1) the total area over which dwellings are spread more or less continuously, (2) the density of dwellings per acre, and (3) the meteorological behaviour of the air as a whole.

*Correlation with extent and density of built-up area.*

If lung cancer is caused in part by atmospheric pollution through smoke from domestic chimneys the rate would be expected to increase as the total number of inhabited dwellings in the vicinity increases, and it should also be positively correlated with the density per acre. Comparative Mortality Ratios (C.M.R.) for males in 1946–49 were as follows :

| | |
|---|---|
| Groups of adjacent towns with over 200,000 occupied dwellings : | |
| London, East Ham, West Ham, Croydon | 156 |
| Birmingham, Smethwick, Walsall, West Bromwich | 134 |
| Manchester, Salford, Stockport | 159 |
| Liverpool, Bootle, Birkenhead, Wallasey | 162 |
| Leeds, Bradford, Halifax | 132 |
| Sheffield, with 124,000 occupied dwellings | 135 |
| Newcastle and Gateshead, with 87,000 occupied dwellings | 114 |
| Aggregate of 6 towns, each with 50,000 to 85,000 occupied dwellings | 113 |
| Aggregate of 3 towns, each with 40,000 to 50,000 occupied dwellings | 107 |
| Aggregate of 12 towns, each with 30,000 to 40,000 occupied dwellings | 104 |
| Aggregate of 13 towns, each with 20,000 to 30,000 occupied dwellings | 100 |
| Aggregate of 29 towns, each with less than 20,000 occupied dwellings | 89 |

Within the groups having less than 50,000 occupied dwellings there was a large range of mortalities for individual towns, explicable by the small numbers of deaths and consequent chance variation, and by special peculiarities. For example the seaside resorts Brighton, Southend, Eastbourne and Southport had rather high rates, but they are dormitory towns and places to which many men retire after working or living in London, Manchester and Liverpool, and are therefore exceptions which would " prove the rule". St. Helens, Warrington, Cardiff and Swansea also had higher rates than their sizes appeared to warrant. There appears to be a general tendency, however, for lung cancer mortality to increase with total number of chimneys until the dwellings exceed 100,000, after which some other factor must be invoked to account for the very high rates in London, Manchester and Merseyside. That other factor could be density per acre, which is 41 in the London area, and 25 in Manchester and Merseyside, compared with 20 in the Birmingham and 11 in the Leeds group. Taking all the County Boroughs separately, there is a correlation coefficient of 0·47 between lung cancer mortality and persons per acre.

Whatever may have been the position about differing completeness of diagnosis and certification 20 years ago, they could hardly now account for such definite relations between lung cancer death rates and the extent and density of built-up areas ; nor can it be supposed that people smoke twice as much tobacco in large towns as in small. The facts, including those brought out in Tables II and III, seem to fit in with the hypothesis that atmospheric pollution by smoke is an important factor on which tobacco smoking is superimposed. The carcinogenic substance, whatever it is, might be common to both kinds of smoke, or these might contain different irritants. The findings of Waller (1952) and of Goulden, Kennaway and Urquhart (1952) on the amounts of bedzpyrene and of arsenic in the air of large towns are of great interest in this connection.

*Wind direction as a possible factor.*

Returning now to Greater London, Clark (1951) has shown that the logarithm of the population density per acre in successive zones diminishes in arithmetic progression on passing outwards from the business centre, and the same is true of other large cities. Supposing domestic smoke to be an important factor in producing lung cancer, if there was no wind at all one would expect to find the highest incidence at the centre, diminishing in all directions outwards. If there is wind and it comes from all directions equally, a more diffused but still symmetrical distribution would be expected, with less contrast near the centre and more near the periphery. However, wind does not blow equally from all directions, and Greenwich records over 12-hour periods throughout the year 1950 show that the wind direction, when analysed into 8 sectors, was S.W. for 35 per cent of the time, W. for 15 per cent, S. for 14 per cent, N.E. for 10 per cent, N.W. for 8 per cent, E. for 7 per cent, N. for 6 per cent, and S.E. for 5 per cent. During half the time, therefore, wind came from one quarter of the circle distributed around W.S.W., whereas from the reverse quarter distributed around E.N.E. the wind blew for only one sixth of the time. The expected effect of that on smoke density would be to shift the maximum some distance from the centre of Greater London in a direction E.N.E., and that is what the map for lung cancer mortality in London County shows, the black patch covering Finsbury, Shoreditch, Bethnal Green, Stepney and Bermondsey. There is also a positive correlation of 0·37 with density per acre in the Metropolitan boroughs, which are of course affected by their own smoke as well as by that blown from other parts of London.

*Atmospheric pollution and tobacco smoking.*

How could this hypothesis be squared with the Medical Research Council figures about tobacco smoking in Greater London ? If 90 per cent of lung cancer in that area was due to tobacco alone, it could not be squared ; but, remembering that about one-third of the female patients with lung cancer had not smoked, the figures are not incompatible if it be supposed that the effects of tobacco and atmospheric pollution are additive. This is easier to perceive in terms of an analogy. Suppose there are set out in a garden 50 jars with capacities ranging from 5 to 10 pints, one-fifth each holding 6, 7, 8 and 9 pints, and one-tenth each holding 5 to 10 pints. Arrange them in 5 rows of 10 each, evenly distributed as to capacity, and suppose that water does not evaporate. During a year leave the first row alone, add 1 pint of water to each jar in the 2nd row, 2 pints to the 3rd row, 3 pints to the 4th row, and 4 pints to the 5th row, and count how many jars have overflowed in each row during the year. Suppose one finds 1 in the first row, 3 in the 2nd, 5 in the 3rd, 7 in the 4th, and 9 in the 5th. If one did not know the capacities of the jars and forgot that it had rained a lot during the year, it would appear that overflow depended entirely on the water added, except that 1 out of 10 overflowed without doing anything. But in actual fact each of the 25 jars which overflowed received 5 pints of rain water, a total of 125 pints, and there were added only 70 pints, so rain was really a bigger factor in producing the overflow than was the water added.

Like all analogies, this will break down if pushed too far, but it shows that the total contribution of tobacco smoking to lung cancer incidence cannot be deduced from the relative frequencies found amongst non-smokers and smokers of 10, 20, 30, 40 cigarettes a day in Greater London alone. It will be necessary to do similar studies amongst residents of rural areas, where it is conceivable that smoking does not begin to show an appreciable relation with incidence of lung cancer until the number of daily cigarettes is at a higher level than appears to be the case in London. In terms of the water jar analogy, if the experiment is repeated where the rainfall is smaller and contributes only 3 pints a year instead of 5, overflow would only begin at the 3rd row and then increase. This would mean, in effect, that smoking is more dangerous to a town dweller than to a country dweller.

This hypothesis is no more than a theory which seems to fit most of the statistical findings so far available. It has not been proved, and may be found later to be untenable. It can be tested, however, and plans are now ready, thanks to the British Empire Cancer Campaign, and its Cheshire and North Wales Group, to collect data during the next five years throughout North Wales and Liverpool Hospital Region in the hope of elucidating this hypothesis about lung cancer at the same time as other field studies are being made concerning pathogenesis of cancer of the stomach and other organs. In that work the hospitals, radium institutes and medical officers of health are co-operating.

Although, as one is continually reminded, statistics in themselves cannot establish causation, and in common with other types of cancer research they often lead up blind alleys, they can start what biochemists and pathologists will finish, and they may even succeed in making sense out of a chaos of isolated findings by other research workers. It seems possible that this may happen sooner for lung carcinoma than for any other form of cancer.

In conclusion, I have to acknowledge my indebtedness to the Registrar-General for access to unpublished details of deaths in 1931–46, and to former colleagues at the General Register Office who helped in their extraction.

### REFERENCES.

Clark, C.—(1951) *J. R. Statist. Soc.*, **114**, 490.
Clemmesen, J.—(1951) *J. nat. Cancer Inst.*, **12**, 1.
*Idem* and Nielsen, A.—(1951) *Brit. J. Cancer*, **5**, 159.
Council for Co-ordination of International Congresses of Medical Sciences.—(1951) Symposium on Geographical Pathology and Demography of Cancer (Session held at Oxford in 1950).
Daff, M. E., Doll, R., and Kennaway, E. L.—(1951) *Ibid.*, **5**, 1.
Doll, R., and Hill, B.—(1950) *Brit. med. J.*, **2**, 739.
Goulden, F., Kennaway, E. L., and Urquhart, M. E.—(1952). *Brit. J. Cancer*, **6**, 1.
Stern, R.—(1842) *Giornali per Servire al Progressi della Pathologia e della Terapeutica*, 2.2.507.
Stocks, P.—(1936) *Ann. Rep. Brit. Emp. Cancer Campgn.*, **13**, 240.—(1937) *Ibid.*, **14**, 198.—(1939) *Ibid.*, **16**, 308.—(1947) ‘ Studies on Medical and Population Subjects No. 1 ; Regional and Local Differences in Cancer Deaths Rates. London (H.M. Stationery Office).
Versluys, J. J.—(1949) *Brit. J. Cancer*, **3**, 161.
Waller, R. E. (1952) *Ibid*, **6**, 8.
Wynder, E. L., and Graham, E. A.—(1950) *J. Amer. med. Ass.*, **143**, 329.